# Investigating the effects of a cryptic splice site in the *En2* splice acceptor sequence used in the IKMC knockout-first alleles

**DOI:** 10.1007/s00335-024-10071-2

**Published:** 2024-10-01

**Authors:** Prerna Nair, Karen P. Steel, Morag A. Lewis

**Affiliations:** Wolfson Sensory, Pain and Regeneration Centre, King’s College London, London, SE1 1UL UK

**Keywords:** Knockout-first, *En2* splice acceptor, Cryptic splice site, IKMC, IMPC, Mutant mouse allele

## Abstract

Targeted mouse mutants are a common tool used to investigate gene function. The International Knockout Mouse Consortium undertook a large-scale screen of mouse mutants, making use of the knockout-first allele design that contains the *En2* splice acceptor sequence coupled to the lacZ reporter gene. Although the knockout-first allele was designed to interfere with splicing and thus disrupt gene function, the *En2* sequence has been reported to be transcribed within the host gene mRNA due to a cryptic splice site within the *En2* sequence which allows splicing to the next exon of the host gene. In some circumstances, this has the potential to permit translation of a mutant protein. Here, we describe our computational analysis of all the mouse protein-coding genes with established knockout-first embryonic stem cell lines, and our predictions of their transcription outcome should the *En2* sequence be included. As part of the large-scale mutagenesis program, mutant mice underwent a broad phenotyping screen, and their phenotypes are available. No wide-scale effects on mouse phenotypes reported were found as a result of the predicted *En2* insertion. However, the *En2* insertion was found experimentally in the transcripts of 24 of 35 mutant alleles examined, including the five already described, two with evidence of readthrough. Splicing from the cryptic splice site also has the potential to disrupt expression of the lacZ reporter gene. It is recommended that mutant transcripts be checked for this insertion as well as for leaky transcription in studies involving knockout-first alleles.

**Supplementary Information:**

The online version contains supplementary material available at 10.1007/s00335-024-10071-2.

## Introduction

The mouse genome is reported to contain approximately 22,000 protein-coding genes (http://www.ensembl.org/Mus_musculus/Info/Annotation, accessed June 2024). To understand the roles of these genes, an international collaboration, the International Knockout Mouse Consortium (IKMC), was undertaken with the goal of creating an extensive library of conditional knockouts in mouse ES cells. The data are available on the website of the International Mouse Phenotyping Consortium (IMPC; https://www.mousephenotype.org) (Groza et al. 2023; Skarnes et al. 2011). As part of the knockout mouse program, mutant mouse lines underwent the same phenotype observation pipeline, which took a broad screening approach with the goal of addressing a wide spectrum of diseases (Birling et al. 2021; White et al. 2013). These mouse mutants have proven to be a highly valuable resource for investigating multiple forms of disease, and there now are over 7,200 peer-reviewed publications citing the IMPC (https://www.mousephenotype.org/data/publications, accessed June 2024).

**Fig. 1:**
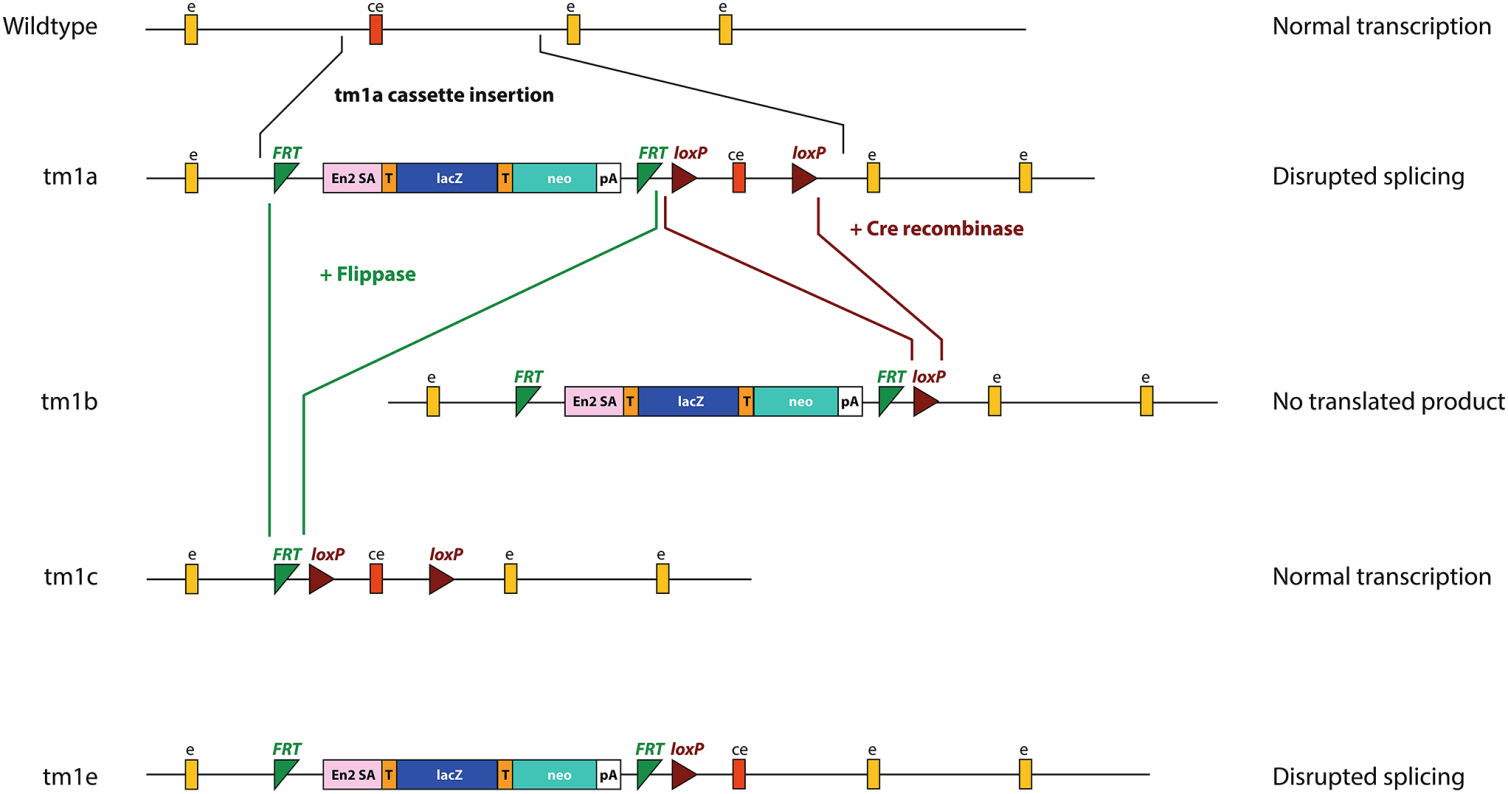
Schematic of the knockout-first allele, and its different versions. Yellow boxes (e) show exons, the red box shows the critical exon (ce), green triangles show FRT sites, and brown triangles show the loxP sites. The main portion of the cassette consists of the lacZ gene (lacZ, blue rectangle), with the *En2* splice acceptor sequence (En2 SA, pink) at the 5’ end, and the neomycin resistance gene (neo, teal rectangle). Both genes have T2A sites (orange, T) at their 5’ ends and the neomycin gene has a polyadenylation site (pA) at the 3’ end; the T2A sites ensure each gene product is translated independently. This is the promoterless cassette; the promoter-driven cassette has an additional loxP site and the beta-actin promoter directly before the neomycin resistance gene, which increases its expression. It also has an internal ribosome entry site at the start of the lacZ gene instead of the first T2A site, a polyadenylation sequence at the end of the lacZ gene, and does not include the second T2A site (Skarnes et al. 2011). The tm1a (knockout-first) allele has the cassette inserted into the intron before the critical exon, leaving the critical exon itself intact. The tm1b (lacZ-tagged deletion) allele can be generated using Cre recombinase (brown lines), and the tm1c conditional allele can be generated using Flippase (green lines). The tm1e (targeted, non-conditional) allele is the same as the tm1a allele but lacks the final loxP site, which is due to a crossover event in the targeted ES cell clone (Skarnes et al. 2011); it does not directly derive from the tm1a allele and cannot be converted to a conditional allele using Flippase

The knockout-first allele was designed to be a flexible approach where multiple types of alleles can be obtained from a single starting allele; the design allows for an attempt to recover gene function once it has already been disrupted (Skarnes et al. 2011; Testa et al. 2004). The starting allele consists of a large disruption cassette which is targeted to a specific intron of a protein-coding gene, chosen to be directly upstream of a “critical” exon. Ideally, a critical exon is one which is present in all transcripts of the gene, which would introduce a frameshift when deleted (i.e. its length is not divisible by 3), and would interrupt a functional domain and occur early in the gene, although for some genes not all of these requirements could be met (Skarnes et al. 2011). The large size of the cassette is predicted to prevent normal splicing, thus interfering with the transcription of the gene (Fig. 1).

The knockout-first targeted mutation allele (tm1a) is the original, starting allele. The number refers to the attempt made by the researchers; so where a second attempt is needed to create the allele, it would be called ‘tm2a’. The knockout-first allele consists of several key components, including a lacZ reporter gene, a neomycin resistance cassette, *FRT* sites, and *loxP* sites (Fig. 1). Several other alleles can be generated from the tm1a allele. The *FRT* sites can be used to convert the knockout-first tm1a allele to a conditional allele (tm1c), where gene function is rescued, using the flippase (FLP) enzyme to remove the large transcription-disrupting cassette (Fig. 1). The *loxP* sites allow recombination when exposed to the Cre recombinase enzyme which deletes the critical exon, resulting in a lacZ-tagged deletion allele (tm1b), which lacks the critical exon entirely (Fig. 1) (Skarnes et al. 2011). The tm1e allele is a targeted non-conditional allele, without conditional potential because it has lost the 3’ *loxP* site (Fig. 1) (https://www.mousephenotype.org/understand/start-using-the-impc/allele-design/). While the tm1e allele is still damaging, it cannot be converted into a tm1b allele, and use of Flippase would simply remove the disruption cassette, rather than creating the tm1c conditional allele.

In the context of this study, the most important aspect of the cassette is a short sequence tagged onto the start of the lacZ gene: the splice acceptor site of the engrailed 2 (*En2*) gene. The *En2* splice acceptor site is 158 bp in length, with the following sequence: *gt*cccaggtcccgaaaaccaaagaagaagaaccctaacaaagaggacaagcggcctcgcacagccttcactgctgagcagctccagaggctcaaggctgagtttcagaccaac**aggt**acctgacagagcagcggcgccagagtctggcacaggagctc (bold marks the cryptic splice donor site discussed below; the canonical *En2* acceptor site at the start of the exon sequence is underlined). This sequence was inserted in order to ensure lacZ was spliced to and transcribed effectively (Gossler et al. 1989; Skarnes et al. 2011).

**Fig. 2:**
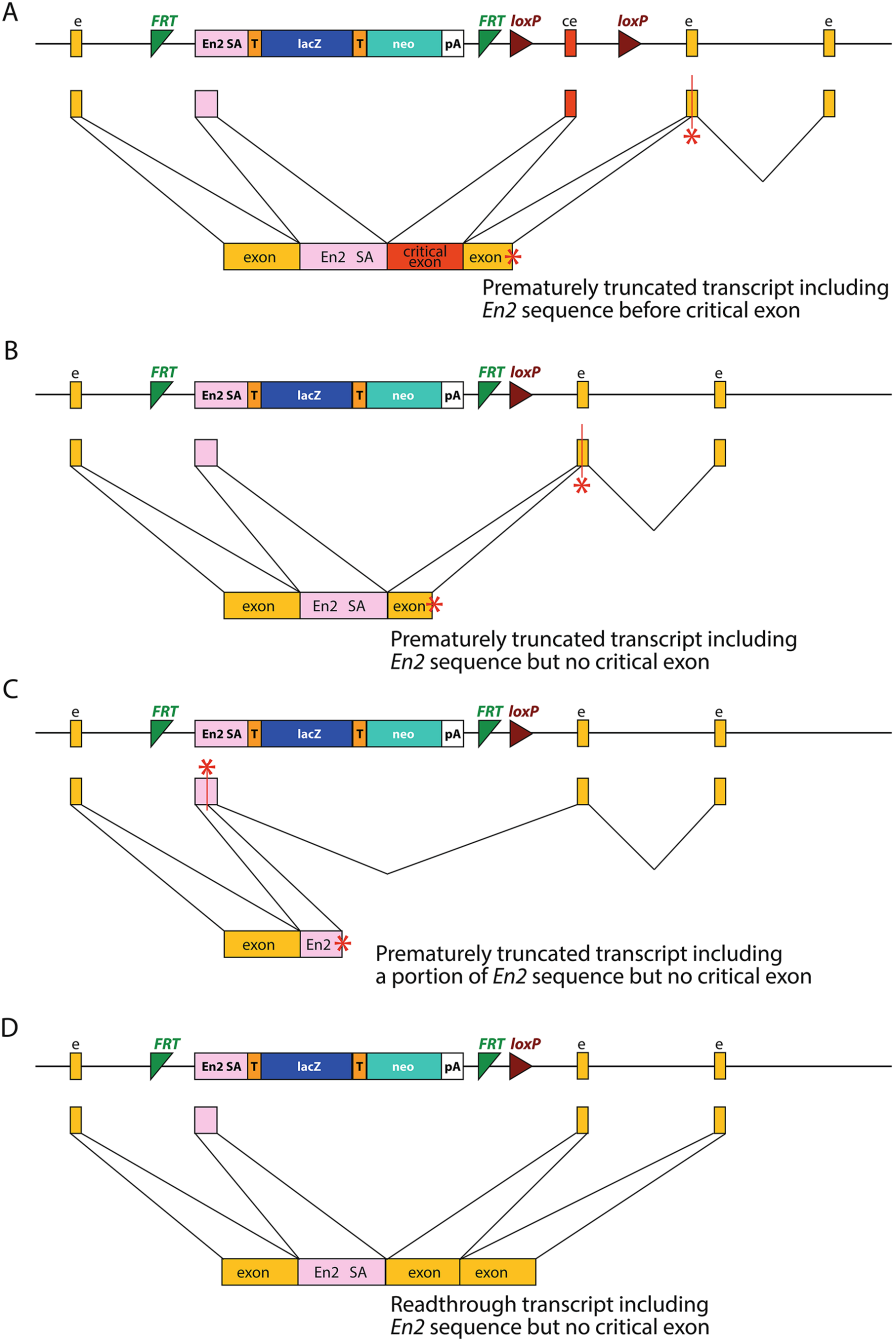
Examples of *En2* splice acceptor sequence inclusion. (**A**) Insertion of the *En2* splice acceptor before the critical exon in a tm1a allele, as seen in *Hnrnpr* (Ghanawi et al. 2021). In this case, the inserted sequence results in a frameshift and a transcript which truncates at a premature stop codon (red asterisk). (**B**) Insertion of the *En2* splice acceptor in place of the critical exon in a tm1b allele, as seen in *Synj2* (Martelletti et al. 2020) and *Whrn* (Ebrahim et al. 2016). In these cases, the inserted sequence is also predicted to result in a frameshift and a truncated protein. (**C**) In some cases, the insertion of the *En2* splice acceptor in place of the critical exon results in a stop codon within the *En2* insertion itself, as seen in *Rhbdf1* (Hosur et al. 2020). (**D**) However, in *Ccdc50* (Lachgar-Ruiz et al. 2023), the inserted sequence maintains the reading frame and results in a mutant protein with a section from the *En2* gene

Mutant mice from the IKMC have been widely used for investigating gene function, and along with the reports describing this research, several studies have reported the detection of a mutant transcript which includes a 115 bp section of the *En2* sequence (gtcccaggtcccgaaaaccaaagaagaagaaccctaacaaagaggacaagcggcctcgcacagccttcactgctgagcagctccagaggctcaaggctgagtttcagaccaac**ag**; the remaining 2 bp of the cryptic splice donor site are shown in bold) (Ebrahim et al. 2016; Ghanawi et al. 2021; Hosur et al. 2020; Lachgar-Ruiz et al. 2023; Martelletti et al. 2020). Both tm1a and tm1b alleles were found to include this *En2* insertion sequence. In the case of the *Hnrnpr* tm1a allele, the *En2* insertion was included immediately before the critical exon (Ghanawi et al. 2021) (Fig. 2A), but in the case of the tm1b alleles reported thus far, the *En2* insertion replaced the critical exon. In some mutants, this is predicted to result in a frameshift, the introduction of a stop codon, and nonsense-mediated decay (*Whrn* (Ebrahim et al. 2016); *Synj2* (Martelletti et al. 2020) (Fig. 2B), and in others, a stop codon is introduced within the *En2* insertion itself (*Rhbdf1* (Hosur et al. 2020) (Fig. 2C). However, in some cases, it could potentially allow readthrough and the generation of a mutant protein (*Ccdc50* (Lachgar-Ruiz et al. 2023) (Fig. 2D).

This unexpected splicing is thought to be due to a cryptic splice site partway through the *En2* sequence, which allows splicing from within the sequence to the next exon of the host gene. The effect of this on transcription will depend on how the *En2* sequence fits into the endogenous mRNA; the result may be a protein with the *En2* splice acceptor site sequence inserted that could be at least partially functional. It is important for researchers, who may be expecting a full knockout of the gene, to know which targeted knockouts may exhibit this behaviour that can result in a different phenotype than the intended knockout allele. We have investigated the potential for these different outcomes in 14,262 knockout-first allele designs and tested for the presence of the insertion in 30 mutant lines in addition to the 5 previously reported. We have also assessed the effect of this unexpected splicing upon the expression of the lacZ reporter gene.

## Methods

### Phase calculations

**Fig. 3:**
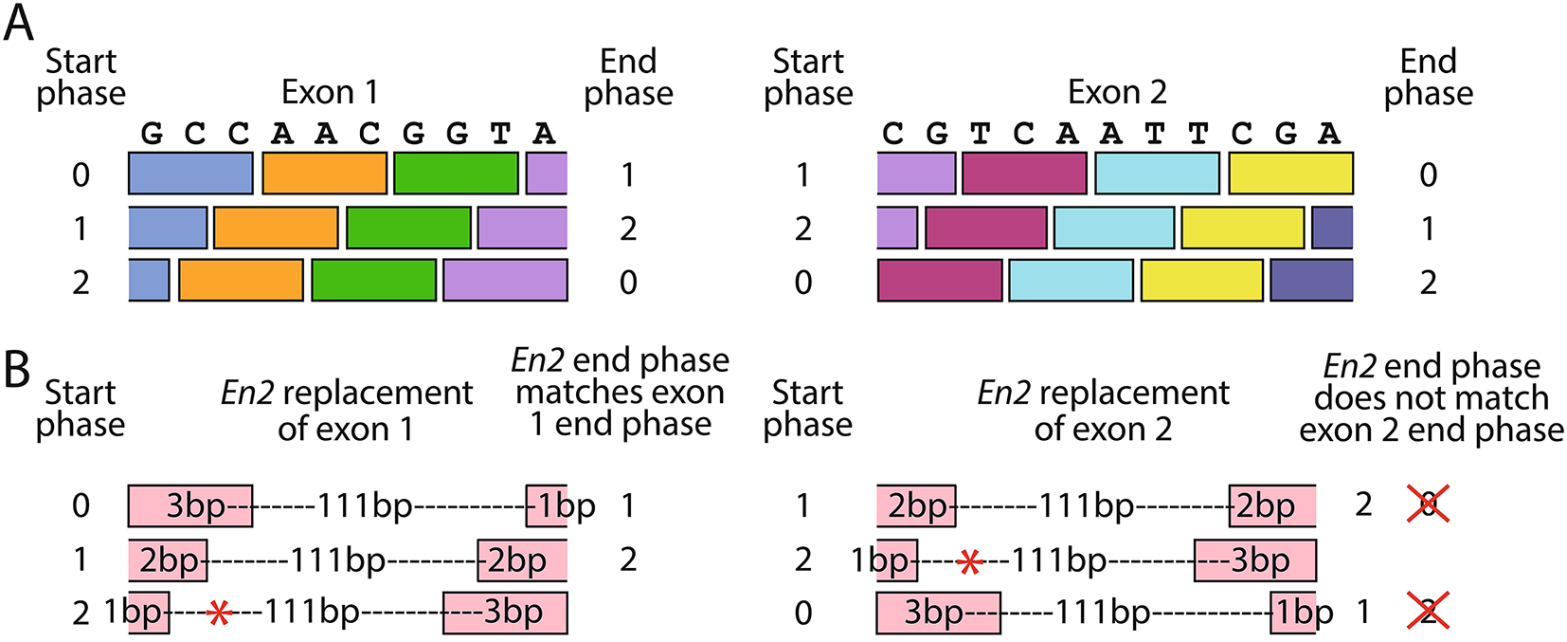
Phase calculations for the *En2* insertion. **A**. Two representative exon sequences are shown on the top of the diagram, 10 and 11 base pairs in length. The coloured boxes show potential codons for the three different start phases, with end phases also shown. The phase number is the start phase of the current exon, as well as the end phase of the previous exon. **B**. Replacing exon 1 with the 115 bp *En2* insertion (pink boxes, below) would permit readthrough if the exon starts in phase 0 or 1, since the end phases of the *En2* insertion match the end phases of exon 1. However, for exon 2, the end phases do not match, so if the exon starts in phases 0 or 1, the result of the *En2* insertion is predicted to be a frameshift. If the *En2* insertion replaces an exon which starts in phase 2, a stop codon is introduced within the *En2* sequence itself (red asterisk), resulting in a truncated transcript. If the previous exon ends in phase 2 with ‘TA’ (as exon 1 in the example), the first ‘G’ of the *En2* insertion would make an extra stop codon at the very start of the insertion, with the same ultimate effect

The effect of the insertion of the *En2* sequence into a transcript depends firstly on whether it is inserted before the critical exon or in place of the critical exon. Because the *En2* sequence up to the cryptic splice site is 115 bp long, which is not a multiple of 3, it will introduce a frameshift if inserted before the critical exon in a knockout-first (tm1a) allele. However, if it replaces the critical exon, its effect depends on the length of the critical exon and the phase in which the *En2* sequence is read. Each exon has a start and end phase, which may be 0, 1, or 2. The start phase refers to the position of the intron/exon boundary within a codon, which corresponds to the number of bases used from the previous exon to form a complete codon. For example, for an exon starting in phase 1, the first two bases of the exon will join the last base from the previous exon to form a codon (Fig. 3A). This also means the end phase of one exon is the same as the start phase of the next exon.

The transcription outcome for each of the 9 potential phase combinations are shown in Table 1. If the critical exon is read starting in phase 2, a TAA stop codon will be transcribed from within the *En2* sequence, starting at base 37. However with the other two potential start phases, if the critical exon ends in the same phase as the inserted *En2* sequence, there could be a readthrough, with the *En2* sequence being incorporated into the transcript in place of the critical exon. If the critical exon end phase differs from that of the *En2* sequence, it would result in a frameshift (Fig. 3B).

**Table 1:** Start-end phase combinations of the critical exon

Start Phase	End Phase	Predicted Effect	Resulting protein sequence
0	0	Frameshift	VPGPENQRRRTLTKRTSGLAQPSLLSSSRGSRLSFRPT(g)
0	1	Readthrough	VPGPENQRRRTLTKRTSGLAQPSLLSSSRGSRLSFRPT(g)
0	2	Frameshift	VPGPENQRRRTLTKRTSGLAQPSLLSSSRGSRLSFRPT(g)
1	0	Frameshift	(gt)PRSRKPKKKNPNKEDKRPRTAFTAEQLQRLKAEFQTN(ag)
1	1	Frameshift	(gt)PRSRKPKKKNPNKEDKRPRTAFTAEQLQRLKAEFQTN(ag)
1	2	Readthrough	(gt)PRSRKPKKKNPNKEDKRPRTAFTAEQLQRLKAEFQTN(ag)
2	0	Stop codon within *En2*	(g)SQVPKTKEEEP*
2	1	Stop codon within *En2*	(g)SQVPKTKEEEP*
2	2	Stop codon within *En2*	(g)SQVPKTKEEEP*

The 9 possible combinations of start and end phases are shown above, along with the predicted effect of the *En2* insertion on the transcript if it replaces the critical exon, and the translation of the inserted sequence. The sequence starting in phase 1 is the EN2 amino acid sequence (amino acids 220–258 in EN2), which includes the first helix of the homeodomain (amino acids 236–292 in EN2) but is otherwise disordered in structure. Brackets and lowercase letters show the DNA sequence of the partial codons which cannot be translated without knowledge of the preceding or following exon sequence. The asterisk indicates the introduced stop codon when starting in phase 2

## Computational analysis

**Fig. 4:**
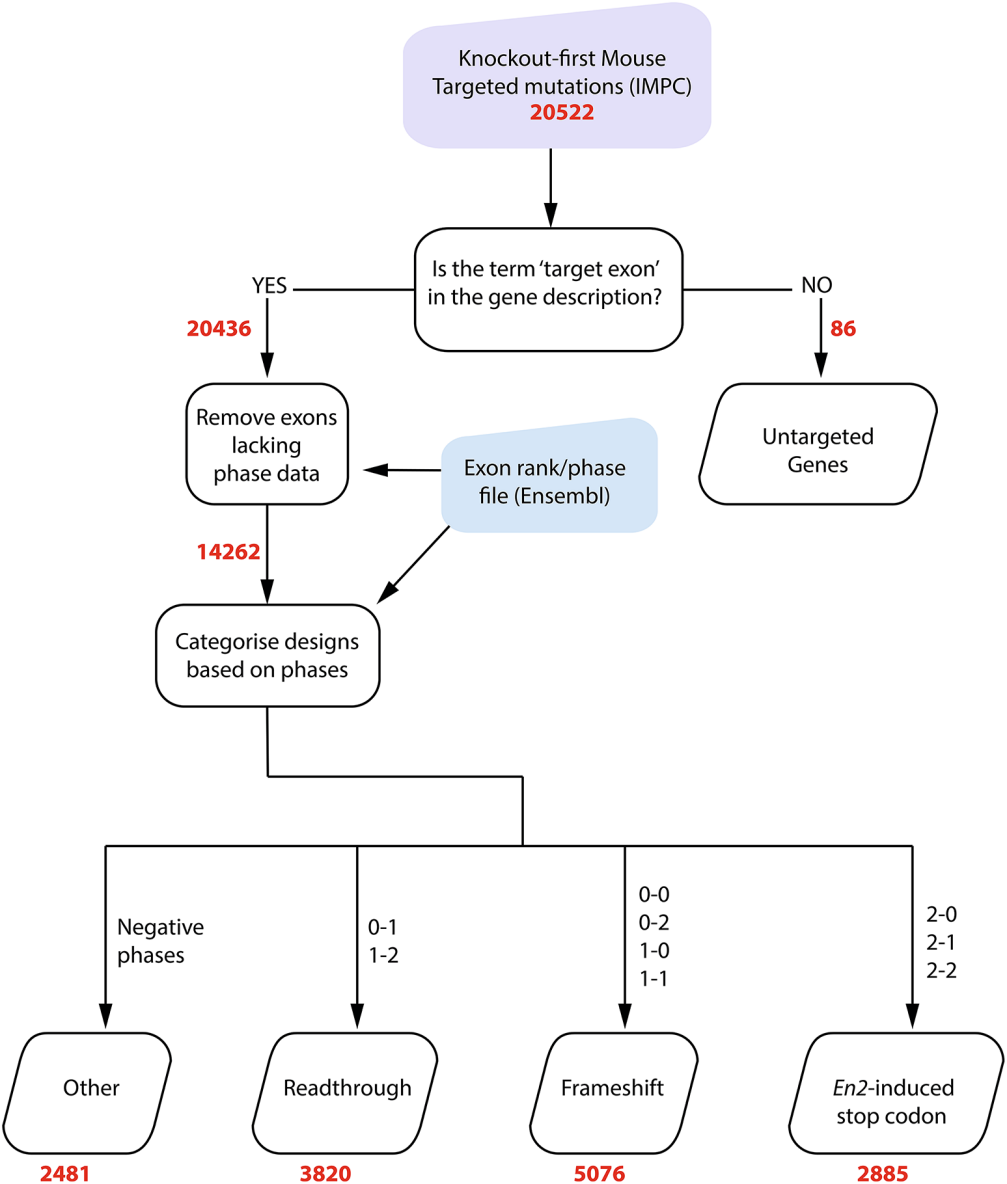
Transcription outcome prediction flowchart. Input files are shown in the coloured shapes, and automated processes are shown in squares. Files that were the output of the script are shown in parallelograms. The numbers (bold red text) show the number of designs that are present at each stage of analysis. The start and end phase combinations of the critical exon(s) are also shown

The online databases Ensembl (http://www.ensembl.org/index.html) and IMPC (https://www.mousephenotype.org/) were used to collect bulk data for computational analysis. All genomic data is from the Genome Reference Consortium Mouse Build 39 (GRCm39) release. Scripts were written in python to parse the files and carry out further analysis (Fig. 4); specifically, to obtain exon phases for the critical exon(s) for each targeted mutation, to classify each mutation according to the phase calculations (Table 1), and to calculate the number and severity of recorded phenotypes. The script is available on GitHub (https://github.com/prernanair/En2-Cryptic-Splice-Site).

### Ethics statement

Mouse studies were carried out in accordance with UK Home Office regulations and the UK Animals (Scientific Procedures) Act of 1986 under UK Home Office licences, and the study was approved by the Wellcome Sanger Institute or the King’s College London Animal Welfare and Ethical Review Bodies. Mice were culled using methods approved under these licences to minimise any possibility of suffering. Mice were group-housed in individually ventilated cages at a standard temperature and humidity and in specific-pathogen-free conditions, with lighting on a 12 h on/12 hours off cycle, and in accordance with the EU Directive 2010/63/EU for animal experiments. Both males and females were used. Samples were collected within a 1.5-hour window from 6 h after lights on.

### Sample collection

Samples from thirty mouse (*Mus musculus*, NCBI Taxon ID 10090) lines representing 27 genes were used to test our predictions (Table 2). Brain tissue was snap-frozen in liquid nitrogen, and inner ear tissue was preserved in RNAlater (Invitrogen, cat.no AM7024) before being frozen.

### RNA extraction, cDNA creation and RT-PCR

Tissue samples were used from previous published and unpublished studies (e.g. (Chen et al. 2024; Ingham et al. 2020; Lachgar-Ruiz et al. 2023). For all samples, RNA was either extracted from brain using TRIzol (Invitrogen, cat no 15596018) or from inner ear tissue using a QIAgen RNEasy kit (cat no 74104) or a Lexogen SPLIT kit (cat no 008.48), all as per the manufacturer’s instructions. cDNA was made using either Superscript II Reverse Transcriptase (Invitrogen, cat no 18064014), Superscript VILO Reverse Transcriptase (Invitrogen, cat no 11766050) or Primerdesign RT nanoscript 2 (cat no RT-premix2-48). The samples used represent a range of ages (Table 2).

**Table 2:** Details of tissues used

Gene	Ensembl ID	Allele	Source tissue	Age
*A430005L14Rik*	ENSMUSG00000047613	tm1a	Brain	Juvenile (P14)
*Amz2*	ENSMUSG00000020610	tm1e	Brain	Juvenile (P14)
*Arsg*	ENSMUSG00000020604	tm1a	Brain	Juvenile (P14)
*Ccdc50*	ENSMUSG00000038127	tm1a	Brain	Adult (P40)
*Cdh23*	ENSMUSG00000012819	tm2a	Brain	Adult (P98-P99)
*Col4a3*	ENSMUSG00000079465	tm1a	Inner ear	Adult (P188)
*Dclk1*	ENSMUSG00000027797	tm1a	Brain	Juvenile (P14), elderly (P404)
*Evi5*	ENSMUSG00000011831	tm1a	Brain	Juvenile (P14)
*Fzd6*	ENSMUSG00000022297	tm2a	Brain	Juvenile (P14)
*Grm8*	ENSMUSG00000024211	tm2a	Brain	Juvenile (P14)
*Klhl18*	ENSMUSG00000054792	tm1a	Brain	Adult (P42)
*Myo19*	ENSMUSG00000020527	tm1a	Inner ear	Adult (P28)
*Myo7a*	ENSMUSG00000030761	tm1a	Brain	Adult (P29)
*Ninl*	ENSMUSG00000068115	tm1a	Brain	Adult (P42-P50)
*Ninl*	ENSMUSG00000068115	tm1b	Brain	Adult (P44-P49)
*Ocm*	ENSMUSG00000029618	tm1e	Organ of Corti	Adult (P54-P56)
*Pex3*	ENSMUSG00000019809	tm1a	Brain	Adult (P28-29)
*Ptprd*	ENSMUSG00000028399	tm2a	Brain	Juvenile (P14)
*Sgms1*	ENSMUSG00000040451	tm1a	Inner ear	Early postnatal (P4)
*Sgms1*	ENSMUSG00000040451	tm1b	Inner ear	Early postnatal (P4)
*Sik3*	ENSMUSG00000034135	tm1a	Brain	Juvenile (P14)
*Spns2*	ENSMUSG00000040447	tm1a	Brain	Adult (P66-P69)
*Spns2*	ENSMUSG00000040447	tm1b	Brain	Adult (P54)
*Srrm4*	ENSMUSG00000063919	tm1e	Organ of Corti	Early postnatal (P4)
*Srsf7*	ENSMUSG00000024097	tm1a	Brain	Adult (P101)
*Tgm6*	ENSMUSG00000027403	tm1a	Brain	Juvenile (P14)
*Usp42*	ENSMUSG00000051306	tm2a	Brain	Adult (P69-P79)
*Wbp2*	ENSMUSG00000034341	tm1a	Brain	Adult (P37)
*Whrn*	ENSMUSG00000039137	tm1a	Inner ear	Adult (P28)
*Zfp719*	ENSMUSG00000030469	tm1a	Inner ear	Juvenile (P14)

P = postnatal day

Primers were designed to amplify from exons before and after the critical exon using Primer3 (Untergasser et al. 2012). Primer sequences are in Online Resource 1. A touchdown PCR protocol was used to amplify cDNA, as follows: 94 °C for 2 min; 94 °C for 30 s; 64 °C for 45 s (decreasing by 0.5 °C per cycle); 72 °C for 45 s; Carry out steps 2–4 16 times in total; 94 °C for 30 s then 55°C for 45 s then 72 °C for 45 s, repeated 21 times in total; 72 °C for 7 min.

PCR samples underwent an enzymatic cleanup using Illustra ExoProStar (Cytiva Life Sciences, cat no GEUS77705) and were sequenced by Source Bioscience (Nottingham, UK). Sequencing reads were aligned and analysed using Gap4 (Bonfield et al. 1995).

## Results

### Categorization into transcription predictions

14,262 allele designs had phase information and were analysed and sorted into categories based on the phase combinations described in Table 1, assuming that the *En2* sequence replaces the critical exon. 2,481 designs were categorized separately into the ‘Other’ category as they had either negative start or end phases, indicating the exon/intron boundary is in the non-coding region of the pre-mRNA (Table 3). These were not analysed further, with the exception of those exons starting in phase 2 and ending in a negative phase, where the inclusion of the *En2* sequence would result in a slightly truncated protein; these were included with the *En2*-induced stop codon counts.

**Table 3:** Number of designs in each predicted effect category

Category	Number of designs	Number of genes
Readthrough	3820	3650
Frameshift	5076	4861
*En2* induced stop codon	2885	2747
Other	2481	2438

228 genes had multiple designs based around different critical exons such that they were placed in more than one category (226 genes had two designs, and 2 genes (*Dock8* and *Tnnc2*) had three designs). The full list of genes analysed and the predictions are in Online Resource 2

## Phenotypic differences

**Fig. 5:**
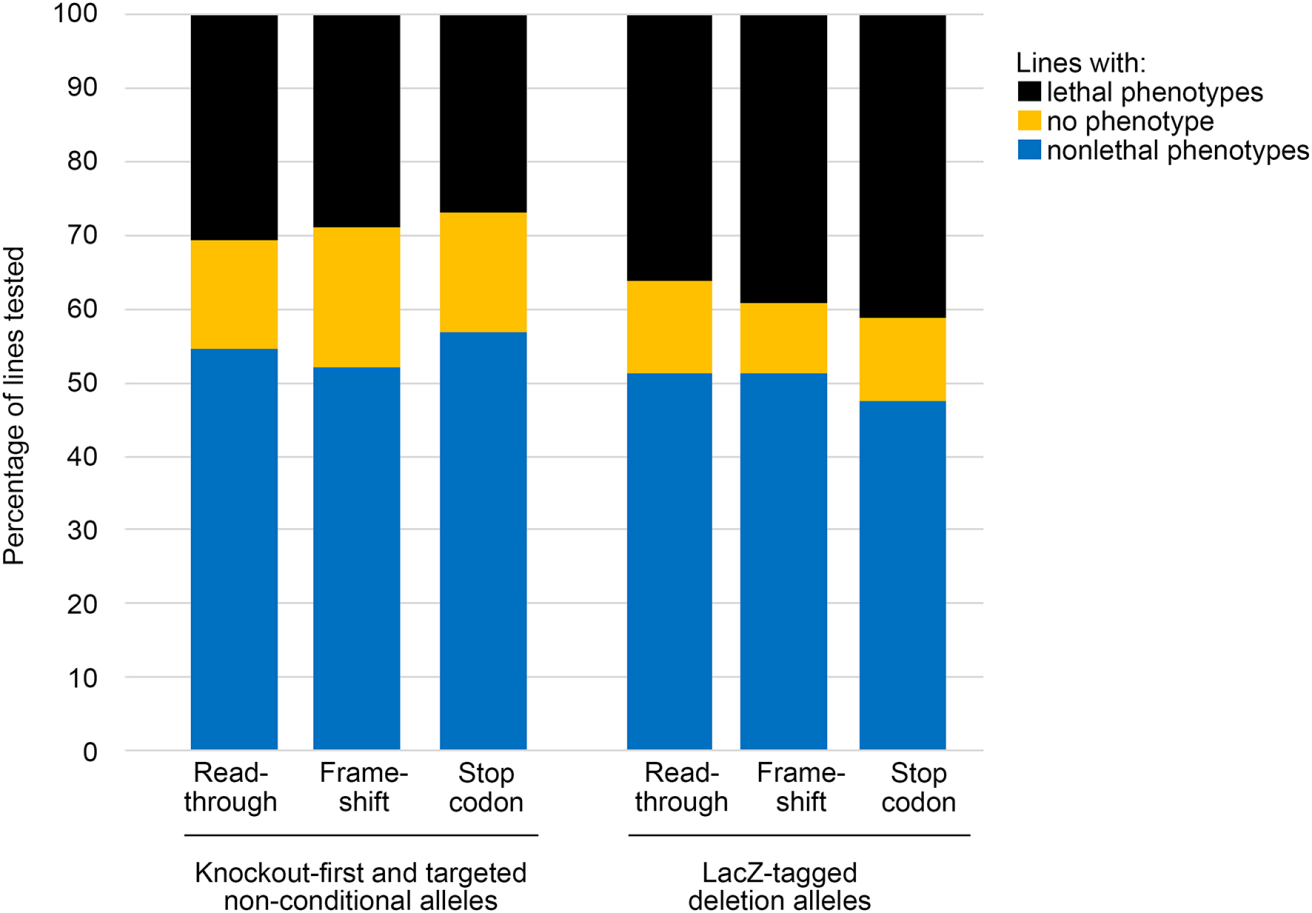
Proportion of tested lines showing lethal, nonlethal and no phenotypes grouped by allele type and transcription outcome prediction. Genes with multiple designs in different categories have been excluded

Data on the phenotypes of mice carrying these alleles were obtained from the IMPC (https://www.mousephenotype.org, (Groza et al. 2023) FTP site (http://ftp.ebi.ac.uk/pub/databases/impc/all-data-releases/latest/results/procedureCompletenessAndPhenotypeHits.csv.gz and http://ftp.ebi.ac.uk/pub/databases/impc/all-data-releases/latest/results/laczExpression.csv.gz, accessed June 2024) as a list of mutant lines and the outcome of tests carried out, including associated Mammalian Phenotype (MP) terms (https://www.informatics.jax.org/vocab/mp_ontology/). Each MP term associated with a mutant mouse line represents a significant difference between control and mutant mice. Significance in the IMPC data is assessed using the OpenStats software package, which was designed for high-throughput phenotypic data (Haselimashhadi et al. 2020). We counted the number of associated MP terms for knockout-first and targeted non-conditional alleles, and for all forms of reporter-tagged deletion allele, and considered the percentage of genes which produced no abnormal phenotype, the percentage of lines with a lethality phenotype, and the average number of abnormal phenotypes per mouse line. None of these showed any notable differences between the different transcription outcome categories (Table 4; Fig. 5).

**Table 4:** IMPC-based analysis of a broad range of phenotypes, categorized by predicted transcription outcomes and by mutant allele type

		Readthrough	Frameshift	*En2* induced stop codon
Starting number of genes		3650	4861	2747
Untested genes (no phenotype data) (% of total)		2592 (71.0%)	3385 (69.6%)	1899 (69.1%)
Knockout-first and targeted non-conditional alleles	Lines tested	335	431	261
	Lines with no phenotype (% of lines tested)	49 (14.6%)	82 (19.0%)	43 (16.5%)
	Lines with lethal phenotypes (% of lines tested)	103 (30.7%)	125 (29.0%)	70 (26.8%)
	Number of phenotypes/tested line	4.04	3.72	3.52
LacZ-tagged deletion alleles	Lines tested	764	1089	616
	Lines with no phenotype (% of lines tested)	96 (12.6%)	105 (9.6%)	69 (11.2%)
	Lines with lethal phenotypes (% of lines tested)	277 (36.3%)	426 (39.1%)	254 (41.2%)
	Number of phenotypes/tested line	4.63	4.94	4.63

Genes with multiple designs in different categories have been excluded from the counts, since there was no way to identify which allele design was used for the phenotyping from the data available

We also checked for the absence of any lacZ expression, but found only 36 genes where there was no expression recorded in any of 27 or more tissues tested out of a total of 2909 genes.

### Testing for the presence of the *En2* insertion in the transcripts of targeted genes

There were thirty-five mutant lines for which data were available, thirty from this study and five previously reported. Twenty-eight were knockout-first (tm1a, tm2a) or targeted non-conditional (tm1e) alleles, and seven were lacZ-tagged deletion (tm1b) alleles. Of the 28 tm1a, tm1e and tm2a alleles, 18 carried the 115 bp *En2* sequence before the critical exon and one (*Col4a3*^*tm1a*^) carried the *En2* sequence in place of the critical exon (Table 5). Of the seven tm1b alleles, five carried the *En2* sequence in place of the critical exon (Table 5). Two of the 35 alleles analysed experimentally showed readthrough or readthrough potential, where the *En2* sequence was seen in place of the critical exon.

**Table 5:** Number of observations of transcripts containing the *En2* sequence before or in place of the critical exon in the thirty-five lines documented

Gene	Allele	Transcripts with *En2* sequence visible…		Number of mice tested			Prediction (if *En2* replaces critical exon)	LacZ expression reported
		Before critical exon	In place of critical exon	Homozygote	Hetero-zygote	Wildtype		
*A430005L14Rik*	tm1a	5		4	1	2	Frameshift	Ingham et al. 2020
*Amz2*	tm1e	2		1	1	1	Readthrough	Ingham et al. 2020
*Arsg*	tm1a	0		1	1	1	Readthrough	
*Ccdc50*	tm1a	0		2	1	1	Readthrough	Reported on IMPC website (tm1b allele)
*Cdh23*	tm2a	2 (hom)		2	2	2	Readthrough	
*Col4a3*	tm1a		1	1		0	Readthrough	Reported on IMPC website (tm1b allele)
*Dclk1*	tm1a	0		3	1	2	Frameshift	Ingham et al. 2020
*Evi5*	tm1a	2 (hom)		3	1	0	Stop codon	
*Fzd6*	tm2a	2		1	1	0	Stop codon	Ingham et al. 2020
*Grm8*	tm2a	0		2		1	Readthrough	
*Hnrnpr*	tm1a	Yes					Frameshift	
*Klhl18*	tm1a	1 (hom)		1	1	1	Stop codon	
*Myo19*	tm1a	0		3		1	Readthrough	
*Myo7a*	tm1a	2		2		1	Frameshift	
*Ninl*	tm1a	0		3	1	2	Frameshift	Reported on IMPC website (tm2a allele)
*Ocm*	tm1e	3		3		2	Frameshift	
*Pex3*	tm1a	0		3	1	2	Stop codon	Kochaj et al. 2022
*Ptprd*	tm2a	2		3		1	Frameshift	
*Sgms1*	tm1a	2		2		2	Stop codon	Chen et al. 2024
*Sik3*	tm1a	2 (hom)		2	1	2	Frameshift	
*Spns2*	tm1a	2		2		0	Frameshift	Chen et al. 2014
*Srrm4*	tm1e	1		2		2	Stop codon	
*Srsf7*	tm1a	2			2	2	Readthrough	
*Tgm6*	tm1a	0		1	2	1	Frameshift	Ingham et al. 2020
*Usp42*	tm2a	3		3		2	Readthrough	Reported on IMPC website
*Wbp2*	tm1a	0		1	1	1	Stop codon	Buniello et al. 2016
*Whrn*	tm1a	2		2		1	Frameshift	Reported on IMPC website
*Zfps719*	tm1a	2		2		2	Readthrough	Reported on IMPC website
*Ccdc50*	tm1b		Yes				Readthrough	Reported on IMPC website
*Ninl*	tm1b		0	3	1	2	Frameshift	Reported on IMPC website (tm2a allele)
*Rhbdf1*	tm1b		Yes				Stop codon	
*Sgms1*	tm1b		3 (hom)	3	2	1	Stop codon	Chen et al. 2024
*Spns2*	tm1b		0		2	2	Frameshift	Chen et al. 2014
*Synj2*	tm1b		Yes				Frameshift	Martelletti et al. 2020
*Whrn*	tm1b		Yes				Frameshift	Ebrahim et al. 2016

*Whrn*^*tm1b*^, *Hnrnpr*^*tm1a*^, *Rhbdf1*^*tm1b*^, *Ccdc50*^*tm1b*^ and *Synj2*^*tm1b*^ are included without numbers, based on previous reports ((Ebrahim et al. 2016; Ghanawi et al. 2021; Hosur et al. 2020; Lachgar-Ruiz et al. 2023; Martelletti et al. 2020). Where a subset of mutant mice were found to contain the *En2* insertion, and both homozygotes and heterozygotes were tested, the genotype of those containing the insertion is included in the third and fourth columns in brackets (*Cdh23*, *Evi5*, *Klhl18*, *Sik3*, and *Sgms1*; in all cases the samples including the *En2* insertion were from homozygous animals). If lacZ expression has been reported, the reference is shown in the final column. For *Ninl*, the expression data comes from the tm2a allele, but the design is identical to the tm1a allele used in this study so this expression has also been included

## Discussion

After testing, and including the alleles already reported (Ebrahim et al. 2016; Ghanawi et al. 2021; Hosur et al. 2020; Lachgar-Ruiz et al. 2023; Martelletti et al. 2020), the *En2* sequence was found to be present in mRNA from 24 of 35 lines tested overall; 19 of 28 knockout-first (tm1a, tm2a) and targeted non-conditional (tm1e) alleles and five of seven lacZ-tagged deletion (tm1b) alleles (Table 5). There were seven cases where a subset of individual animals exhibited the *En2* insertion, suggesting that its inclusion depends on more than just the host gene and chosen site for the cassette insertion. This may be a source of variation between mutant animals carrying the same allele. It should be noted that for these experiments, the tissue used was from either the brain or the inner ear, and different results may be obtained from other tissues due to differences in tissue-specific expression and splicing.

For most of the knockout-first and targeted non-conditional alleles where the *En2* insertion was observed, it was inserted before the critical exon, thus inducing a frameshift, so the ultimate purpose of the allele, transcriptional disruption of the host gene, would still be achieved. However, we observed one example (*Col4a3*) where the *En2* insertion replaced the critical exon, in which case the ultimate outcome depends on the start and end phases of the critical exon, as it does for the lacZ-tagged deletion (tm1b) alleles. For those alleles where the *En2* sequence replaces the critical exon and where readthrough is predicted to occur, a malformed protein may result. This malformed protein may be a functional null, but may also be a hypomorph, or even exhibit gain-of-function effects, and thus the phenotypes observed may not be the result of a loss of protein function. This is a separate phenomenon to “leaky” transcription, which occurs when some full-length mRNA is transcribed with correct splicing despite the allele being designed to prevent it. Leaky transcription has been reported from several knockout-first alleles (Ingham et al. 2020; Martelletti et al. 2020; White et al. 2013) and is a known potential issue with the targeted mutation allele design.

No quantitative analysis of mRNA level was done in this study, so the overall effects of the *En2* insertion on transcription levels have not been determined. However, a number of tm1a, tm2a, tm1e and tm1b alleles (such as *Sgms1*^*tm1a*^, *Sgms1*^*tm1b*^, *Evi5*^*tm1a*^, *Amz2*^*tm1e*^, and *Ptprd*^*tm2a*^) have been reported to result in downregulation in homozygous mice (Chen et al. 2024; Ingham et al. 2020). All these alleles had some evidence of the *En2* insertion in mutant mice (Table 5).

Despite the apparent prevalence of the *En2* insertion in these mouse mutants, we did not observe any major differences in the occurrence of preweaning lethality, or the number of phenotypes per mouse line in the three transcription outcome categories from the IMPC data (Table 4; Fig. 5). This suggests that even when the *En2* insertion permits readthrough of the mutant transcript, the majority of these alleles will still result in a less functional protein. However, that does not rule out the possibility of an ameliorating effect on a minority of readthrough alleles. For example, *Frmd5*^*tm1a*^ mice exhibit preweaning lethality with incomplete penetrance, but there is no lethality phenotype recorded for the *Frmd5*^*tm1b*^ allele. The *En2* insertion in *Frmd5* is predicted to result in a readthrough (Online Resource 2), which may explain the unexpected loss of the lethality phenotype in the tm1b allele. However, there are other reasons this can occur, including reinitiation from a downstream start codon (as seen in *Rhbdf1*^*tm1b*^ (Hosur et al. 2020), which emphasises the importance of verifying the transcriptional and translational outcomes of each mutant allele used in a study. It should also be noted that approximately 70% of the genes in each transcription outcome category had no available phenotype data, and that mutant phenotypes may be missed if the relevant organ systems or life stages are not tested. The IMPC is a broad phenotyping screen, but it does not cover everything.

We did observe differences between the knockout-first and targeted non-conditional alleles (tm1a, tm1e) and the lacZ-tagged deletion alleles (tm1b). The percentage of lines with lethality is higher for lacZ-tagged deletion alleles, and they also have a higher average number of phenotypes per line, while a lower percentage of lines carrying lacZ-tagged deletion alleles show no phenotype. This suggests that leaky transcription may be rescuing phenotypes in the knockout-first and targeted non-conditional lines. The overall count of phenotypes per line is similar to that observed by Dickinson et al. (Dickinson et al. 2016).

The function of the *En2* sequence in the cassette is to enable splicing to and transcription of the lacZ reporter gene in the time and location in which the host gene would normally be expressed. If splicing takes place between the *En2* cryptic splice site and the next splice acceptor site in the host gene, transcription of the lacZ gene could potentially be disrupted. Such splicing was detected in 24 of 35 alleles tested in this study and others (Table 5), and is independent of the outcome category (readthrough, frameshift or introduced stop codon). However, the presence of the *En2*-included mutant transcript does not mean that the lacZ fusion transcript is not also present, and for 19 of the 35 alleles tested, lacZ expression has been reported either in peer-reviewed publications or by the IMPC (Table 5). We further investigated this using the reported lacZ expression data from the IMPC, and found 36 genes out of 2909 where none of the 27 or more tissues tested exhibited any lacZ expression. However, these genes may be specifically expressed at a life stage or in a tissue which wasn’t investigated, which means it isn’t possible to say from this analysis whether loss of lacZ expression is a problem for this small subset of genes.

This study highlights the importance of confirming the nature of a mutation before conducting experiments that rely on it. Suitable methods to do this include sequencing cDNA made using RNA extracted from the relevant tissue, and Western blots using antibodies against different regions of the protein, if they are available, in order to confirm protein size. With all mutant mouse ES cells created by the IKMC having undergone the same design pipeline, they are all susceptible to the same potential for inclusion of the 115 bp *En2* sequence. It is recommended that when researchers work with knockout-first alleles and alleles derived from them, they assess the transcription outcome prior to interpreting their experiments. A knockdown instead of a knockout of transcription is a more common outcome to consider, but readthrough leading to an abnormal mRNA and potentially an abnormal protein, as described here, is an additional consideration.

## Electronic supplementary material

Below is the link to the electronic supplementary material.

Supplementary Material 1

Supplementary Material 2

Supplementary Material 3

## Data Availability

All data supporting the findings of this study are available within the paper and its Supplementary Information. The python script used for the analysis described in this paper is available at https://github.com/prernanair/En2-Cryptic-Splice-Site.
