# Supplementary material for: Investigating the effects of a cryptic splice site in the En2 splice acceptor sequence used in the IKMC knockout-first alleles
Source: Mamm Genome. 2024 Oct 1;35(4):633–44. doi: 10.1007/s00335-024-10071-2 (PMC11522132; doi:10.1007/s00335-024-10071-2)
Supplement: Supplementary file 3 — Supplementary Material 3 [file 335_2024_10071_MOESM3_ESM.docx]

**Online Resource 1.** Table with sequences of primers used for short-range PCR and sequencing. All primer sequences are shown 5’-3’.

**Online Resource 2.** This table shows the transcription outcome predictions for 14262 allele designs. Where a gene has multiple designs in different categories, the presence of a duplicate is marked. The *En2*-induced stop codon category, where the start phase is phase 2, thus resulting in a stop codon being read partway through the *En2* sequence, includes 239 genes where the end phase is negative, meaning the critical exon is the final exon, so the result should be a transcript truncated close to the 3' end. These are marked "truncated" in addition to "*En2*-induced stop codon". The critical exon IDs are the IDs of the first and last exon to be targeted. Any intermediate exons are not included.
